# Diagnostic value of plasma C-type natriuretic peptide levels in determination of the duration of mesenteric ischaemia

**DOI:** 10.5830/CVJA-2014-033

**Published:** 2014

**Authors:** Sinan Demirtas, Oguz Karahan, Suleyman Yazici, Orkut Guclu, Ahmet Caliskan, Orhan Tezcan, Celal Yavuz

**Affiliations:** Medical School of Dicle University, Department of Cardiovascular Surgery, Diyarbakir, Turkey; Medical School of Dicle University, Department of Cardiovascular Surgery, Diyarbakir, Turkey; Medical School of Dicle University, Department of Cardiovascular Surgery, Diyarbakir, Turkey; Medical School of Dicle University, Department of Cardiovascular Surgery, Diyarbakir, Turkey; Medical School of Dicle University, Department of Cardiovascular Surgery, Diyarbakir, Turkey; Medical School of Dicle University, Department of Cardiovascular Surgery, Diyarbakir, Turkey; Medical School of Dicle University, Department of Cardiovascular Surgery, Diyarbakir, Turkey

**Keywords:** C-type natriuretic peptide, mesenteric ischaemia, ischaemia duration

## Abstract

**Objective:**

Mesenteric arteries release C-type natriuretic peptide (CNP), which hyperpolarises vascular smooth muscle. We measured the levels of this peptide after inducing mesenteric ischaemia over a series of time intervals, so as to determine its predictive value in demonstrating the severity of ischaemia in a rat model.

**Methods:**

A total of 32 rats were allocated to four groups containing eight rats each. Basal CNP reference levels were measured in the control group, which was not exposed to any intervention. In groups I, II and III, mesenteric ischaemia was induced over three, six and nine hours, respectively, and plasma CNP levels were measured afterwards. Mesenteric ischaemia was induced by clamping the superior mesenteric artery.

**Results:**

In comparison with the controls (2.38 ± 0.18 pg/ml), CNP levels were relatively lower in group I (2.54 ± 0.42 pg/ml). However, significant increases in plasma CNP levels were observed over longer periods of ischaemia in group II, at 5.23 ± 0.22 pg/ml, and in group III, at 6.19 ± 0.67 pg/ml (*p* < 0.05). A significant direct relationship was determined between plasma CNP levels and prolonged intervals of mesenteric ischaemia (*R* = 0.56, *p* < 0.001).

**Conclusion:**

Measuring plasma CNP levels in patients with acute mesenteric ischaemia may be beneficial in estimating the time period over which the ischaemic injury has occurred.

## Abstract

Acute mesenteric ischaemia (AMI) causes significant morbidity and mortality if not promptly diagnosed and treated. If medical interventions are delayed, the patient may sustain serious ischaemic injury leading to bowel necrosis, so large segments of bowel may require surgical resection. Often these patients have poor clinical outcomes and suffer from complications such as malnutrition.^1^,^2^ Mesenteric ischaemia makes up 0.1% of all hospital admissions.^1^ Even though technological advances have been made in diagnostic laboratory and imaging techniques, AMI remains fatal in 60% of patients diagnosed with this condition.^1^,^3^

Scientists have been investigating whether there are specific sensitive biomarkers that may indicate the presence of AMI.^2^,^4^ Several endothelial markers have been identified as putative biomarkers that may reveal the severity and duration over which mesenteric ischaemia has been sustained.^5^ However, markers that are effective enough for use in clinical practice have yet to be identified.

Natriuretic peptides, namely atrial natriuretic peptide (ANP), brain natriuretic peptide (BNP), and C-type natriuretic peptide (CNP) function in maintaining fluid and electrolyte balance as well as blood vessel tone. CNP is released by vascular endothelial cells, and this biomarker’s function in influencing vascular tone has been investigated.^8^,^7^ It has been hypothesised that CNP is an endothelium-derived hyperpolarising factor (EDHF) that specifically affects the degree of resistance in the mesenteric arteries.^8^ In this study, we aimed to investigate plasma CNP levels during early and advanced stages of mesenteric ischaemia so as to determine whether CNP levels are a good indicator of severity of AMI in a rat model.

## Methods

The study protocol was created in accordance with the Animal Welfare Act and the Guide for the Care and Use of Laboratory Animals created by the university ethics committee. The rats were obtained and housed in the laboratory of the University’s animal production unit. They were maintained in a controlled environment with 12-hour light–dark cycles, and the cages were kept at a constant humidity of 50 ± 5% and temperature of 22 ± 2°C.

A total of 32 male Sprague-Dawley rats between the ages of eight and 12 weeks and weighing 230 ± 30 g (mean ± standard deviation) were randomly allocated to four different groups. The induction of sedation was achieved with an intraperitoneal injection of 130 mg/kg of ketamine (Ketalar, Pfizer) and 20 mg/kg of xylasine (Rompun, Bayer). Sedation was maintained with 50 mg/kg of ketamine hydrochloride so that the animals remained under anaesthesia during blood collection and superior mesenteric artery clamping.

Blood samples were obtained from the control group to determine basal CNP levels. A simple laparotomy was performed on the rats in groups I, II and III in order to clamp the superior mesenteric artery (SMA) and artificially create mesenteric ischaemia. The SMA remained clamped for three hours in group I, six hours in group II, and nine hours in group III. Blood samples were collected from the animals after the designated duration of induced mesenteric ischaemia without declamping, and then they were sacrificed. Several animals died during the procedure, including one in group II and three in group III, and they were subsequently excluded from the study. Plasma CNP levels were measured from the collected blood samples.

Biochemical analysis was as follows. Blood collection tubes containing citrate were used, and after the samples were obtained they were centrifuged at 4 000 rpm at 4°C for 10 minutes. The centrifuged samples were then transferred into Eppendorf tubes for storage at –80°C.

Commercially available radioimmunoassay kits (RIA) (C-type natriuretic peptide-22, Phoenix Pharmaceuticals, Belmount, CA, USA) were used to determine plasma CNP levels. One millilitre of plasma was eluted with a 1-ml volume of 60% acetonitrile mixed in a 1% trifluoracetic acid (TFA) solution for the solidphase extraction step, as previously described by del Ray *et al.*^7^

After the remaining product was dissolved in 300–500 μl of assay buffer, 100 μl of the resulting mixture was used to perform the immunometric assay.^7^ The average CNP recovery was calculated to be 74.8%.

## Statistical analysis

Statistical calculations were performed with the SPSS software (SPSS version 15.0 for Windows, SPSS Inc., Chicago, IL USA). Data were expressed as the mean ± one standard deviation (SD). The Kolmogorov–Smirnov test was used to assess whether the data conformed to a normal distribution. A *p*-value < 0.05 was considered statistically significant. Significant differences between group means were assessed with one-way analysis of variance (ANOVA). Tukey’s honest significant difference (HSD) was used as a post hoc test.

## Results

In the control group, the mean plasma CNP level was 2.54 ± 0.42 pg/ml. A slight decrease in CNP level was observed in group I relative to the controls following three hours of induced mesenteric ischaemia [2.38 ± 0.18 pg/ml (*p* = 0.085)]. However, mean CNP levels were dramatically increased in group II (5.23 ± 0.22 pg/ml) compared to the controls and group I following six hours of mesenteric ischaemia (*p* = 0.001). Average CNP levels were even higher in group III (6.19 ± 0.67 pg/ml) relative to the controls and group I (*p* = 0.000) and group II (*p* = 0.036).

**Fig. 1.**
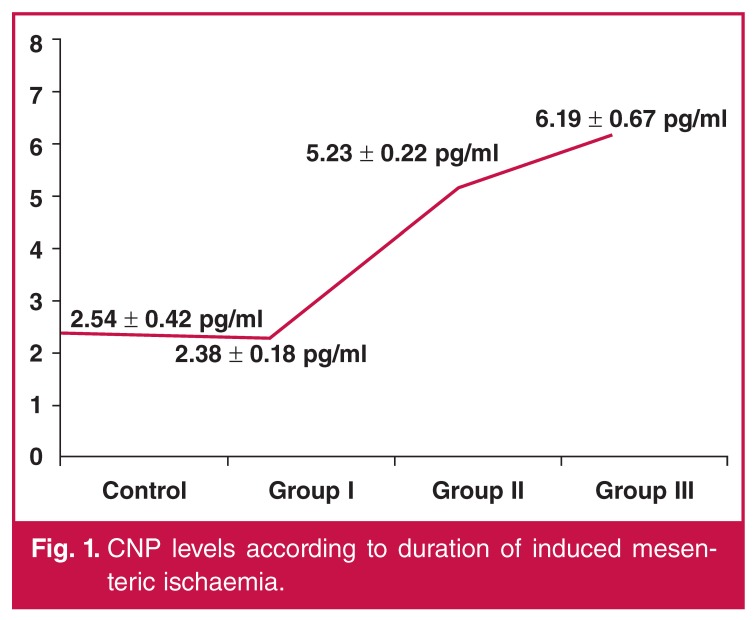
CNP levels according to duration of induced mesenteric ischaemia.

There was a significant positive correlation between plasma CNP levels and longer durations of induced mesenteric ischaemia (*R* = 0.56, *p* < 0.001). The CNP levels observed in each experimental group are summarised in Fig. 1.

## Discussion

The findings of this study indicate that plasma CNP levels were relatively low during the initial stages of mesenteric ischaemia. However, CNP levels quickly elevated in response to longer durations of sustained ischaemic injury. These findings are promising because CNP levels may allow one to differentiate between early and late mesenteric ischaemia.

The initial reduction in CNP levels during the early hours of mesenteric ischaemia may have been due to systemic CNP regulatory pathways. On the other hand, elevated plasma CNP levels during the sixth and ninth hours of induced mesenteric ischaemia may signify delayed mesenteric endothelial resistance or a response compounded by progressively worsening mesenteric ischaemia.

CNP was first isolated from blood collected from the brain and was subsequently categorised into the natriuretic peptide family, which contains three molecules that have a particular 22-amino acid structure.^9^ In later studies, it was reported that CNP may also be isolated from plasma samples obtained from the colon, lung, heart and kidneys.^9^

CNP is a unique endogenous ligand for natriuretic peptide B receptor (NPR-B) and is upregulated by transforming growth factor-β, which is an important vascular remodelling factor.^9^,^10^ NPR-B is located on vascular smooth muscle and modulates vascular tone.^9^,^11^

CNP inhibited proliferation of endothelial and vascular smooth muscle cells in *in vitro* studies.^12^ Additionally, CNP demonstrated anti-atherogenic properties via p-selection suppression, which regulates the recruitment of leukocytes and platelet–leukocyte transmission.^12^

It has been reported that CNP is released from endothelial cells in rat mesenteric vessels and activates endothelium-derived hyperpolarising factor (EDHF). EDHF then triggers potassium channel opening and NPR-B activation so that mesenteric vascular smooth muscle cells will hyperpolarise and relax.^13^ Despite the important role that CNP plays in mesenteric vessel tone, the effects of CNP have not been previously studied in the setting of mesenteric ischaemia.

CNP produced anti-fibrotic and anti-proliferative effects via inhibition of cultured fibroblasts, and reduced tissue growth factor-1 (TGF-1)-induced collagen production in cultured fibroblasts.^14^ Recent reports have suggested that CNP has cardio–renal protective effects via these humoral mechanisms in the setting of stress injury, with suppression of pro-fibrotic processes and a protective function.^14^-^16^

Furthermore, CNP has local regulatory functions via the vascular renin–angiotensin system. CNP inhibits the vasoconstrictor impact of angiotensin 1. Additionally, recent reports suggest that CNP is an endogenous regulator of vascular ACE activity. Higher CNP levels were demonstrated both in renal failure patients who were on haemodialysis therapy and in cardiac failure patients.^15^,^16^

In a recent study it was reported that CNP lacked renal action but led to vasodilatation and inhibition of growth.^19^ These data indicate that CNP is a non-cardiac regulator hormone that regulates vascular tone according to cardio–renal interactions via different mechanisms, such as the vascular renin–angiotensin system.^15^-^17^

Natriuretic peptides are potent vasodilators during hypoxic conditions. For example, Klinger *et al.* reported pulmonary vessel vasodilation in response to natriuretic peptides in rats adapted to hypoxic environments.^9^ Similarly, Zhao *et al.* described the possible use of natriuretic peptides in maintaining pulmonary vascular homeostasis in hypoxic patients.^18^

Hobbs *et al.* studied CNP in an experimental model of myocardial ischaemia–reperfusion and found that CNP had protective vasorelaxation properties.^19^ Ahluwalia *et al.* demonstrated that hypoxia might directly induce the release of CNP so that vascular homeostasis is maintained.^20^

It has been reported that CNP may contribute to the regulation of blood flow with decreasing perfusion pressure and also reduce the oxidative damage after reperfusion in ischaemic conditions.^19^ Additionally, it was hypothesised that CNP was up-regulated in the presence of nitric oxide (NO) synthase inhibition for compensation of the protective role of NO.^19^

In another study, it was shown that CNP led to an increment in NO stimulation and suppression of the neo-intimal hyperplasia and inflammatory process in an experimental carotid injury model.^21^ Chun *et al.* demonstrated that oxidative stress could modulate the endothelium-derived vasoactive substances such as CNP.^22^ Yamahara *et al.* claimed that CNP enhanced angiogenesis in ischaemic conditions in their experimental model.^23^ All these studies identified a range of cellular and vascular interactions that may clarify the role of elevated CNP levels due to oxidative stress during mesenteric ischaemia after reperfusion.

## Conclusion

CNP appears to regulate blood flow in the mesenteric vascular bed. Clinically monitoring CNP levels may be useful in estimating the duration over which the patient has sustained mesenteric ischaemia and the severity of the injury due to acute mesenteric artery occlusion. However, the exact mechanism of the interaction between CNP and the mesenteric vessels must be further elucidated in future clinical studies.
